# High prevalence of atrial conduction abnormalities in Lewy body disease – a marker of cardiac complications?

**DOI:** 10.1007/s11357-024-01479-4

**Published:** 2025-01-10

**Authors:** Keivan Javanshiri, Alexander Siotis, Isak Heyman, Mattias Haglund

**Affiliations:** 1 Division of Pathology, Department of Clinical Sciences Lund, Lund University, Lund, Sweden; 2 Department of Clinical Sciences Helsingborg, Lund University, Helsingborg, Sweden; 3 Cognitive Disorder Research Unit, Department of Clinical Sciences Malmö, Lund University, Malmö, Sweden

**Keywords:** Neurocardiology, Dementia, Atrial cardiomyopathy

## Abstract

Increasing evidence suggests that Lewy body disease (LBD) is associated with clinically important cardiac complications, including sick sinus syndrome, atrial fibrillation and sudden cardiac death. The high prevalence of sick sinus syndrome and atrial fibrillation in LBD suggests the presence of disease-related atrial conduction disorders. To explore whether LBD is associated with atrial conduction disorders, electrocardiographic (ECG) P wave parameters were analyzed in a cohort of LBD patients (n = 74), using age-matched Alzheimer’s disease (AD) patients (n = 25) as controls. P wave terminal force in V1 and P wave duration were found to be significantly greater in the LBD group than in the AD group. In addition, 43 (58%) individuals in the LBD exhibited pathological P wave terminal force (> 4000 µV*ms) vs 3 (12%) in the AD group, and 60 (81%) in the LBD group exhibited pathological P wave duration (≥ 120 ms), vs 13 (52%) in the AD group. The difference could not be explained by atrial fibrillation or atrial enlargement on echocardiogram. The clinical significance of pathological P wave parameters in LBD is unknown, but their presence suggests an atrial cardiomyopathy that could be due to cardiac alpha-synuclein deposition, autonomic dysfunction, or a combination thereof. Future research is warranted to elucidate whether this proposed disease-related atrial cardiomyopathy is related to cardiac alpha-synuclein deposition, whether it is causally related to cardiac complications in LBD, and whether pathological P wave parameters hold potential as a screening tool for cardiac complications in LBD.

## Introduction

Cardiac manifestations are common in Lewy body disease (LBD), and the classification “neurocardiological disorder” has been proposed [1]. Apart from the well-known orthostatic hypotension, prior research has indicated a high prevalence of subclinical sinus node dysfunction (SND) [2] and sick sinus syndrome (SSS) [3] in LBD as well as a near ubiquitous involvement of the heart in the form of alpha-synuclein deposition in cardiac tissue [4, 5]. This raises the possibility that these depositions could, if present in or near the sinus node, affect atrial depolarization and thereby cause SND and subsequently SSS.

SSS poses a particular challenge to the clinician in LBD, as these patients are known to often have manifestations of autonomic dysfunction such as orthostatic hypotension and suffer falls as a consequence. SSS may hypothetically contribute to this phenomenon in two ways: Firstly, by causing syncope due to bradycardia or asystole itself, secondly due to absent compensatory pulse increase upon standing (neurogenic orthostatic hypotension) causing failure to increase cardiac output and thereby affecting cerebral perfusion. Thus, symptoms of SSS may be simplistically attributed to hypotensive spells, which means that potentially treatable causes of falls and impaired quality of life go undetected [6]. Inability to regulate heart rate, manifesting as neurogenic orthostatic hypotension, is generally considered a consequence of cardiac sympathetic denervation, a well-known manifestation of LBD [7].

The atria of the heart undergo numerous structural and functional alterations during healthy aging and disease, which often affect the function of the sinus node as it is located in the right atrium. Hypertension and atrial fibrillation (AF) are associated with atrial enlargement, and atrial enlargement has a mutually perpetuating relationship with AF [8, 9]. Age-related degeneration of the electrical conduction system can cause SND and as a consequence progress to SSS, which is widely considered a disease of the elderly [10].

Historically, most electrocardiographic (ECG) research on atrial pathology has been conducted with a view to predicting AF, but increasing understanding of atrial pathology in the absence of AF has prompted interest in the concept of “atrial cardiomyopathy”, a term used to describe structural or electrophysiological atrial alterations believed to be a consequence of aging and hypertension [11]. Several ECG markers have been developed in order to diagnose atrial cardiomyopathy and they are currently undergoing clinical validation [12]. P wave duration has been shown to be longer in patients with SSS than in controls [13], and increased P wave terminal force in lead V1 (PTFV1) (the product of the duration and the amplitude of negative terminal P wave deflection in lead V1) has been associated with left atrial enlargement and left ventricular fibrosis [14], but not atrial fibrosis [15]. Furthermore, abnormal P wave parameters have been linked to dementia [16] and increased mortality [17], though the nature of this relationship (causation or correlation) has not been established.

If P wave parameters on standard 12-lead ECGs can be used to identify patients at risk of SND, the index of suspicion for underlying SSS might be higher in a patient with recurrent falls, increasing the likelihood of timely diagnosis (for example by ambulatory ECG monitoring) and treatment (by removing/reducing pharmaceutical agents with negative chronotropic effects or by pacemaker implantation). Additionally, sympathetic and parasympathetic blockade exert different effects on P wave morphology [18], raising the possibility that P wave parameters could be used to screen for autonomic dysfunction.

The present study was conducted to explore whether LBD is associated with abnormal P wave parameters suggestive of atrial conduction disorders. Previously published P wave parameters [12] were therefore compared in a cohort of LBD patients and a control group of patients with Alzheimer’s disease (AD).

## Materials and methods

### Study subjects and clinical data

Cases were included retrospectively from a previously described cohort [19] based on the presence of an interpretable ECG obtained in sinus rhythm from the regional MUSE NX, 10.1.5.20087 system (GE Healthcare, Wauwatosa, WI, USA). The last recorded interpretable ECG was used for analysis. Clinical characteristics were obtained from medical records. AF was considered present if confirmed on an ECG or there was a diagnosis of AF in the clinical records. Echocardiography reports were collected, if available, from the clinical records to identify presence of atrial enlargement (noted dichotomously as present or absent).

### P wave parameters

The following P wave parameters were assessed and measured retrospectively with a digital caliper in × 4 magnification according to the definitions of the International Society of Electrocardiology consensus paper: P wave duration (measured in lead II, pathological if ≥ 120 ms), P wave area (measured in lead II, pathological if ≥ 4 ms*mV), P wave voltage (measured in lead I, abnormal if ≤ 0,1 mV), PTFV1 (measured in lead V1, pathological if > 4000 µV*ms)) and interatrial block (IAB) as previously defined [12].

### Ethics

The study was approved by the Regional Ethical Review Board, Lund University (now The Swedish Ethical Review Authority), reference numbers 944–2017 and 2019–00051.

### Statistics

Differences between groups were assessed using Mann–Whitney u test or Fisher’s exact test, as appropriate. To assess P wave parameter rater reliability, 20 randomly chosen cases were assessed by two raters (MH and AS) and the intraclass correlation coefficient (ICC) including their 95% confidence intervals were calculated (two-way, agreement) and classified according to Koo et al. [20]. All calculations were made using R version 4.4.1.

## Results

### Background data

In total, 99 patients were included in the study, 74 LBD patients and 25 AD patients. Background information is shown in Table 1. Briefly, age at death and age at last recorded ECG were not different between groups, whereas the LBD group was predominantly male, and the AD group was predominantly female. No significant difference was observed between groups in terms of the prevalence of documented AF (16% in the LBD group and 12% in the AD group; p = 0.754) or heart rate (79/min in the LBD group and 76/min in the AD group; p = 0.317). The prevalence of type 2 diabetes and hypertension was similar in both groups. Concomitant medication is shown in Table 2. The only significant difference was a greater use of anti-Parkinsonian medication in the LBD group.

**Table 1:** Background data on study subjects

	Number of subjects	Gender distribution	Age at death (SD)	Age at study ECG	Heart rate at study ECG (SD)	Atrial enlargement on echocardiogram	Atrial fibrillation	Hypertension	Type 2 diabetes
Lewy body disease	74	45 M / 29F	77.0 (7.7)	74.8 (7.2)	78.7 (13.8)	6/24	12/74	36/70	5/53
Alzheimer's disease	25	8 M / 17F	78.2 (10.2)	75.4 (10.3)	75.8 (15.4)	4/7	3/25	10/19	2/17

**Table 2:** Concomitant medication used at last follow-up. 10 patients had missing data on concomitant medication or had no prescription medication

	Lewy Body Disease	Alzheimer's disease	*p*-value
Acetylcholine esterase inhibitor	24/67 (36%)	8/22 (36%)	1.000
Memantine	28/67 (42%)	4/22 (18%)	0.072
Antidepressive therapy	26/67 (39%)	9/22 (41%)	1.000
Neuroleptics	10/67 (15%)	3/22 (14%)	1.000
Anti-Parkinsonian therapy	24/67 (36%)	0/22 (0%)	< 0.001
Antiepileptic therapy	5/67 (7%)	1/22 (5%)	1.000
ACE inhibitor or Angiotensin Receptor Blocker	12/67 (18%)	2/22 (9%)	0.503
Betablocker	9/67 (13%)	7/22 (32%)	0.062

### Rater reliability

The results from the ICC calculations are shown in Table 3. Rater agreement was “moderate” for P wave duration, interatrial block and P wave area, “good” for PTFV1 and “excellent” for P wave amplitude.

**Table 3:** Interrater reliability assessed by intraclass correlation coefficient (ICC two-way, agreement model)

	ICC	95% CI
P wave terminal force in V1	0.842	0.721–0.997
P wave amplitude lead I	0.943	0.867–1.000
P wave duration lead II	0.53	0.366–0.739
Interatrial block degree	0.51	N/A (ordinal)
P wave area lead II	0.700	0.490–0.932

### P wave parameters

P wave parameters are shown in Table 4. P wave duration and PTFV1 were significantly greater in the LBD group compared to the AD group (131 vs 122 ms; p = 0.002 and 4505 vs 2562 µV*ms; p < 0.001). PTFV1 was pathological in 43/74 (58%) of LBD patients and 3/12 (12%) of AD patients (p < 0.001), whereas P wave duration was pathological in 60/74 (81%) LBD patients and in 13/25 (52%) AD patients (p = 0.008). The other p wave parameters did not differ significantly. Patients with a history of AF (15/99) had significantly longer P wave duration (140 vs 127 ms; p = 0.008) and a trend towards greater PTFV1 (4855 vs 3865 µV*ms; p = 0.11). The other parameters did not differ between the groups (data not shown). Post-hoc analyses excluding patients on betablockers yielded similar results (PTFV1 4451 vs 2760 µV*ms (p = 0.003) and P wave duration 129 vs 123 ms (p = 0.024)), as did analyses excluding patients on acetylcholine esterase inhibitors, memantine and anti-Parkinsonian medications (data not shown).

**Table 4:** P wave parameters

		Mean P wave terminal force (SD)	Mean P wave duration (SD)	Mean P wave voltage (SD)	Mean P wave area (SD)	Interatrial block stage (SD)
Lewy body disease		4505 µV*ms (2056)	131 ms (16)	0.066 µV (0.023)	6.287 ms*mV (2.715)	1.86 (1.1)
Alzheimer's disease		2562 µV*ms (1268)	122 ms (16)	0.060 µV (0.031)	5.684 ms*mV (2.700)	1.44 (1.0)
P-value for difference		< 0.001	0.002	0.181	0.299	0.109

Interatrial block stage was defined as 0: normal P wave duration; 1: P wave duration > 120 ms; 2: Biphasic P wave in III; 3: Biphasic P wave in III and aVF; 4: Biphasic P wave in III, aVF and II

### Echocardiography data

Atrial enlargement was noted in 10 of 31 echocardiogram reports, with a non-significantly lower prevalence in the LBD group (6/24 (25%) vs 4/7 (57%); p = 0.17). Patients with atrial enlargement on echocardiogram had lower P wave voltage in lead I (0.049 vs 0.071 µV; p = 0.038), the other P wave parameters did not differ significantly between patients with normal and enlarged atria.

Patients with confirmed normal atrial size (n = 21, of which 18 LBD and 3 AD), had similar numerical differences in PTFV1 and P wave duration (5360 vs 3724 µV*ms and 132 vs 125 ms) compared to the whole study cohort.

## Discussion

This is the first study to suggest that LBD, when compared to AD, is associated with a particularly high rate of pathological P wave parameters and thus atrial cardiomyopathy. Combined with previous data suggesting a high prevalence of SSS [2, 3] and AF in LBD [21, 22], the presence of cardiac alpha-synuclein deposition in the hearts of the vast majority of LBD patients [4, 5], the results of this study suggest the presence of an LBD associated atrial cardiomyopathy that potentially could contribute to the challenging disease phenotype with cardiovascular complications and frequent falls.

The existence of a specific disease-related atrial cardiomyopathy is further suggested by markedly pathological PTFV1 and P wave duration despite the facts that 1) no significant difference in the prevalence of atrial enlargement between LBD and AD patients was observed, and 2) patients with normal atrial size showed similar differences of P wave parameters as in the cohort as a whole. Left atrial enlargement has in previous studies been associated with both atrial fibrillation as well as diastolic dysfunction [23, 24]. However, contrary to what might have been expected, there was a trend towards lower rates of atrial enlargement in the LBD group, 6/24 (25%) vs 4/7 (57%) in the AD group (p = 0.17), despite a significantly higher rate of pathological P wave parameters. The small and non-significantly greater prevalence of AF in the LBD group, 16% vs 12% in the AD group, p = 0.754) is unlikely to completely explain the marked discrepancy in PTFV1 and P wave duration. These data suggest that the greater PTFV1 and longer P wave duration in LBD patients are due to a higher prevalence of underlying atrial cardiomyopathy rather than a direct association with atrial fibrillation or atrial enlargement. The cause of this suspected LBD associated atrial cardiomyopathy is unknown.

It is possible that the cardiac denervation often reported in LBD [25] affects P wave parameters as a sign of atrial cardiomyopathy. P wave duration can be affected by autonomic tone [18], and LBD is strongly associated with autonomic dysfunction [26]. Animal data suggest that P wave morphology is affected by irritant inhalation, and that this effect is modulated by sympathetic as well as parasympathetic blockade [27]. This hypothesis would attribute atrial cardiomyopathy in LBD to aberrations in the brain–heart axis affecting the autonomic nervous system, but other mechanisms are conceivable such as direct effects on atrial tissue due to neuroinflammation and release of cardiotropic cytokines, which may play a role in cardiac tissue remodeling [28]. Furthermore, the role of neuroinflammation could potentially be both direct and indirect, as autonomic dysfunction could in turn be a consequence of tissue injury mediated by inflammation.

Alternatively, the higher prevalence of pathological P wave parameters and thus atrial cardiomyopathy could be due to degenerative change in the atrial conduction system, e.g. by cardiac alpha-synuclein deposition, which is near ubiquitous in LBD [4, 5].

Thus, while the present study shows a greater extent of abnormal P wave parameters in LBD compared to AD, it is not possible to conclude whether these are due to extrinsic (e.g. due to autonomic dysfunction and/or denervation potentially mediated by neuroinflammation) or intrinsic (e.g. due to cardiac structural alterations such as alpha-synuclein deposition or cytokine-mediated remodeling) factors, or a combination.

It can be argued that the greater PTFV1 and P wave duration in LBD than in AD might not be related to the neurocognitive disorder but due to other cardiac disease. However, we have previously shown that the burden of cardiovascular disease and risk factors are very similar in LBD and AD, as opposed to vascular dementia [19, 29]. The similar cardiovascular disease burden previously reported justifies the choice of AD patients as the comparator group, as this decreases the risk of confounding by atrial enlargement due to pre-existing cardiovascular disease. Indeed, very similar rates of type 2 diabetes and hypertension were observed in this cohort. Furthermore, the groups were well-matched in terms of age at the analyzed ECG recording.

In terms of study limitations, the P wave duration did not show a particularly high interrater reliability, which may cast doubt on the robustness of the data. While this is an important concern, P wave duration is difficult to measure reliably on routine ECGs, as indicated by several studies with ICCs in the similar range as reported in the present study [30–32]. The ICC for PTFV1 was, on the other hand, quite convincing at 0.842, which is considered “good” [20]. Furthermore, the fact that P wave duration was found to be greater in the group with documented AF can be interpreted as a validation of the P wave duration measurements. An additional limitation is the uneven gender balance. However, P wave duration and PTFV1 do not exhibit significant gender differences on a group level [33]. The total number of echocardiographies (n = 31), particularly in the AD group, was low (n = 7). Though it can be argued that the subset of patients that underwent echocardiography was the group most likely to have underlying heart disease, undiagnosed conditions of relevance to P wave parameters cannot definitively be excluded.

There was a statistical trend (0.062) towards less use of betablocker therapy in the LBD group versus the AD group, and numerically greater use of angiotensin-converting enzyme inhibitors or angiotensin receptor blocker therapy. Hypothetically, this might be due to lower tolerability of betablocker therapy in LBD because of its negative chronotropic effect being particulary deleterious in a population with atrial cardiomyopathy, but the available data do not allow definitive conclusions. It should be highlighted that despite numerically greater use of betablocker therapy in the AD group, the between-group difference in PTFV1 and P wave duration was robust to exclusion of patients on various classes of concomitant medication (including betablocker therapy).

The clinical significance of these P wave abnormalities is unclear. However, since a previous study has shown a high prevalence of bradycardia and SSS in patients with LBD that underwent ambulatory ECG monitoring despite having no prior symptoms indicative of bradyarrhythmia [2], it is intriguing to speculate that P wave parameters could potentially be used to identify patients with LBD-related atrial cardiomyopathy, to identify candidates for future trials of targeted SSS screening and management. Alternatively, if the pathological P wave parameters are caused by aberrations in autonomic tone (sympathetic or parasympathetic), routine ECG may hold promise as a simple and universally available marker of autonomic dysfunction.

In conclusion, this study suggests the existence of an LBD associated atrial cardiomyopathy, which may be involved in the well-known cardiac complications of LBD. Future research should explore whether any particular P wave parameter can be used to predict clinical SSS, cardiac denervation (as assessed on MIBG scintigraphy) and relevant clinical manifestations, such as orthostatic hypotension.

## Data Availability

The data underlying this study are not openly available, but non-identifiable study information can be provided by the corresponding author on reasonable request.
